# Non-invasive sampling in Itatiaia National Park, Brazil: wild mammal parasite detection

**DOI:** 10.1186/s12917-020-02490-5

**Published:** 2020-08-17

**Authors:** Laís Verdan Dib, João Pedro Siqueira Palmer, Camila de Souza Carvalho Class, Jessica Lima Pinheiro, Raissa Cristina Ferreira Ramos, Claudijane Ramos dos Santos, Ana Beatriz Monteiro Fonseca, Karen Gisele Rodríguez-Castro, Camila Francisco Gonçalves, Pedro Manoel Galetti, Otilio Machado Pereira Bastos, Claudia Maria Antunes Uchôa, Laís Lisboa Corrêa, Augusto Cezar Machado Pereira Bastos, Maria Regina Reis Amendoeira, Alynne da Silva Barbosa

**Affiliations:** 1 Department of Microbiology and Parasitology, Laboratory of Parasitology, Federal Fluminense University, Biomedical Institute, Professor Hernani Mello Street, São Domingos, Niterói, Rio de Janeiro 24210-130 Brazil; 2 Statistics Laboratory, Mathematics and Statistics Institute, Fluminense Federal University, Rua Professor Marcos Waldemar de Freitas Reis s/n, bloco G, Gragoatá campus, Niterói, RJ 24210-201 Brazil; 3 Department of Genetics and Evolution, Laboratory of Molecular Biodiversity and Conservation, Federal University of São Carlos, Washington Luis highway, km 235, São Carlos, São Paulo 13565-905 Brazil; 4 Laboratory of Toxoplasmosis and Other Protozoan Diseases, Oswaldo Cruz Foundation (Fiocruz, Rio de Janeiro), Oswaldo Cruz Institute, Avenue Brazil, 4365, Manguinhos, Rio de Janeiro, 21040-360 Brazil

**Keywords:** Gastrointestinal parasites, Wild animals, Coproparasitologic, Trichology, DNA sequencing

## Abstract

**Background:**

Non-invasive sampling through faecal collection is one of the most cost-effective alternatives for monitoring of free-living wild mammals, as it provides information on animal taxonomy as well as the dynamics of the gastrointestinal parasites that potentially infect these animals. In this context, this study aimed to perform an epidemiological survey of gastrointestinal parasites using non-invasive faecal samples from carnivores and artiodactyls identified by stool macroscopy, guard hair morphology and DNA sequencing in Itatiaia National Park. Between 2017 and 2018, faeces from carnivores and artiodactyls were collected along trails in the park. The host species were identified through macroscopic and trichological examinations and molecular biology. To investigate the parasites, the Faust, Lutz and modified Ritchie and Sheather techniques and enzyme immunoassays to detect *Cryptosporidium* sp. antigens were used.

**Results:**

A total of 244 stool samples were collected. The species identified were *Chrysocyon brachyurus*, *Leopardus guttulus*, *Canis familiaris*, *Cerdocyon thous*, *Puma yagouaroundi*, *Leopardus pardalis*, *Puma concolor* and *Sus scrofa*. There were 81.1% samples that were positive for parasites distributed mainly in the high part of the park. Helminths, especially eggs of the family Ascarididae, were more frequently detected in carnivore faeces (70.9%). Protozoa, especially *Cryptosporidium* sp., represented the highest frequency of infection in artiodactyl faeces (87.1%). This zoonotic protozoon was detected in eight mammalian species, including in a wild boar. High values of structural richness and Shannon and Simpson diversity indices were observed for the parasites, especially in the faeces of *C. brachyurus*. Significant differences in parasite diversity were observed between wild and domestic animals, such as *C. brachyurus* and *C. familiaris*, respectively, and between taxonomically distant species, such as *C. brachyurus* and *S. scrofa*. The highest values for parasite similarity were found among the species that frequented similar areas of the park, such as *C. brachyurus* and *L. guttulus*.

**Conclusions:**

The animals and parasite infections were identified through the combination of three techniques. High frequency parasite structures were diagnosed. Zoonotic protozoa were found and mainly occurred in samples from introduced species.

## Background

Over the years, wild mammalian fauna have been declining around the world for different reasons, including vehicle fatalities, agricultural frontier expansion, pasture formation, deforestation, environmental pollution and fur trafficking [1, 2]. Another factor that may result in declines in mammalian fauna is the parasite load of different aetiological agents, such as gastrointestinal parasites. These agents infect a myriad of hosts and make important alterations to community structure, directly impacting biodiversity and ecosystem dynamics [3]. The susceptibility of hosts to these infections is related to their phylogenetic proximity, body morphology and dietary habits [4]. Parasitism may result in weight loss, metabolic imbalance, reproductive disorders, anaemia, dehydration, foetal malformations, locomotor injuries, and even death among wild animals [5, 6]. When parasitized, many wild mammals end up presenting behavioural and functional alterations in their niche [7–9]. Therefore, studies on the prevalence of parasites in both wild and sympatric domestic animals are important for better understanding the possible effects on wildlife, the parasitic distribution dynamics among the hosts and possible parasitic sources for fauna.

There are three main types of sampling for wild mammal studies: destructive sampling from dead animals; non-destructive sampling from captured animals; and non-invasive sampling, in which samples, such as traces in the environment, loose hair or feathers, faeces and other remnants, are obtained without catching or handling the animal [10, 11]. It is worth mentioning that non-invasive sampling has become an alternative strategy for species monitoring and conservation, especially for those species with low population density and elusive nocturnal habits, such as carnivores [12]. Through non-invasive faecal sampling, it is possible to obtain data about biodiversity, including identification of the species inhabiting a region, their diet composition and their role in the ecosystem, as well as information about potentially infectious gastrointestinal parasites of these animals [13].

Brazil harbours high mammal biodiversity that includes different species of carnivores and artiodactyls [14], many of which are endangered [15]. However, very little is known about the parasite distribution in Brazilian wild mammal fauna, especially in conservation areas [16]. To obtain reliable information about the mammalian fauna from non-invasive samples and to correlate it to parasite biodiversity, it is essential to precisely identify the host, which is possible through the association of faecal macroscopy, trichology of guard hairs (syn: overhairs), and DNA sequencing.

The use of morphological analysis to study faeces from free-living wild animals is very important since it serves as an initial screening of the samples to be collected, allowing reliable host taxonomic classification at the order level [17]. On the other hand, the trichology of mammalian guard hairs can provide specific information about the host species by associating the guard hair colour pattern (macroscopic guard-hair morphology) and the analysis of its cuticle and medullary designs (microscopic guard-hair morphology). Moreover, DNA sequencing is widely used in studies of free-living wild mammals; similar to trichology, it can also identify the host species.

To avoid possible ecological imbalances, it is important to perform constant monitoring of the wildlife in conservation areas. In this context and while any parasitological study was performed with mammals in the first Brazilian national park, this study aimed to perform an epidemiological survey of gastrointestinal parasites in non-invasive faecal samples from carnivores and artiodactyls identified by stool macroscopy, guard-hair trichology and DNA sequencing in Itatiaia National Park (PNI).

## Results

### Host identification

Three identification techniques, macroscopy, trichology and DNA sequencing, were used to analyse 244 faecal samples (S1, supplementary material and Table 1). It was possible to confirm eight mammal species, which included seven carnivores and one artiodactyl. The host species were identified for 180 of the samples. However, the three techniques did not achieve the same results in 110 of the samples for which the species were identified.

**Table 1:** Hosts classification based on the association of faecal macroscopy, guard hair trichology and DNA sequencing

**Taxonomy**	**Macroscopy**	**Trichology**	**DNA sequencing**	**Pearson’s correlation coeficiente (*ρ*)**	**Total fecal samples (*n*** **= 244)**
**Order Carnivora**					**168 (68.8%)**
**Family Canidae**					**112 (45.9%)**
*Chrysocyon brachyurus*					97 (39.7%)
	Order Carnivora	*C. brachyurus*	*C. brachyurus*	^c^	42
	Order Carnivora	Family Mustelidae	*C. brachyurus*	0.95	11
	Order Carnivora	Family Mephitidae	*C. brachyurus*	0.95	2
	Order Carnivora	*C. brachyurus*	Low quality gene sequence	0.97	6
	Order Carnivora	Family Canidae	*C. brachyurus*	^c^	1
	Order Carnivora	Absent guard hair	*C. brachyurus*	0.95	24
	Family Canidae	*C. brachyurus*	*C. brachyurus*	^c^	3
	Family Felidae	*C. brachyurus*	*C. brachyurus*	0.99	7
	Family Felidae	Family Canidae	*C. brachyurus*	–	1
*Canis familiaris*					13 (5.3%)
	Order Carnivora	Family Canidae	*C. familiaris*	^c^	8
	Order Carnivora	*Procyon cancrivorus*	*C. familiaris*	–	1
	Order Carnivora	Family Mephitidae	*C. familiaris*	–	1
	Order Carnivora	Absent guard hair	*C. familiaris*	–	2
	Family Canidae	Family Canidae	*C. familiaris*	^c^	1
*Cerdocyon thous*					2 (0.8%)
	Order Carnivora	*C. thous*	*C. thous*	^c^	1
	Order Carnivora	Absent guard hair	*C. thous*	–	1
**Family Felidae**					56 (22.9%)
*Leopardus guttulus*					52 (21.3%)
	Family Felidae	*L. guttulus*	*L. guttulus*	^c^	3
	Family Felidae	*L. guttulus*	Low quality gene sequence	0.97	1
	Family Felidae	Absent guard hair	*L. guttulus*	0.94	24
	Family Felidae	Family Canidae	*L. guttulus*	0.94	6
	Family Felidae	*Procyon cancrivorus*	*L. guttulus*	0.94	2
	Family Felidae	Family Mustelidae	*L. guttulus*	0.94	2
	Family Felidae	Family Mephitidae	*L. guttulus*	0.94	5
	Order Carnivora	Absent guard hair	*L. guttulus*	–	7
	Order Carnivora	*L. pardalis*	*L. guttulus*	–	1
	Order Carnivora	*P. yagouaroundi*	*L. guttulus*	–	1
*Puma yagouaroundi*					2 (0.8%)
	Family Felidae	*P. yagouaroundi*	Low quality gene sequence	0.68	2
*Leopardus pardalis*					1 (0.4%)
	Order Carnivora	*L. pardalis*	Low quality gene sequence	–	1
*Puma concolor*					1 (0.4%)
	Order Carnivora	Família Mustelidae	*P. concolor*	–	1
**Order Artiodactyla**					**31 (12.7%)**
**Family Suidae**					**12 (4.9%)**
*Sus scrofa*					12 (4.9%)
	Order Artiodactyla	Absent guard hair	*S. scrofa*	–	1
	Order Artiodactyla	Order Artiodactyla	*S. scrofa*	^c^	11
**Taxonomy**	**Macroscopy**	**Trichology**	**DNA sequencing**	**Total fecal samples (*n*** **= 244)**	
**Order Carnivora**				**168 (68.8%)**	
Order Carnivora US^a^				45 (18.4%)	
	Order Carnivora	*L. guttulus*	*C. familiaris*	1	
	Order Carnivora	*L. pardalis*	*C. brachyurus*	2	
	Order Carnivora	*P. yagouaroundi*	*C. brachyurus*	1	
	Order Carnivora	Absent guard hair	Low quality gene sequence	9	
	Order Carnivora	Family Mephitidae	Low quality gene sequence	1	
	Order Carnivora	*C. brachyurus*	*L. guttulus*	5	
	Order Carnivora	*Leopardus wiedii*	*C. thous*	1	
	Order Carnivora	*Nasua nasua*	Low quality gene sequence	1	
	Order Carnivora	Family Mustelidae	Low quality gene sequence	1	
	Order Carnivora	Family Canidae	Low quality gene sequence	4	
	Family Felidae	*C. brachyurus*	*L. guttulus*	1	
	Family Felidae	Family Canidae	Low quality gene sequence	3	
	Family Felidae	*Nasua nasua*	Low quality gene sequence	1	
	Family Felidae	Family Mustelidae	Low quality gene sequence	3	
	Family Felidae	Absent guard hair	Low quality gene sequence	6	
	Family Felidae	Family Mephitidae	Low quality gene sequence	4	
	Family Felidae	*C. brachyurus*	Low quality gene sequence	1	
**Order Artiodactyla**				**31 (12.7%)**	
Order Artiodactyla US^b^				19 (7.8%)	
	Order Artiodactyla	Absent guard hair	Low quality gene sequence	6	
	Order Artiodactyla	Order Artiodactyla	Low quality gene sequence	13	

^a^ Order Carnivora Unidentified Species; ^b^ Order Artiodactyla Unidentified Species. ^c^ Total agreement among the three identification techniques

Therefore, Pearson’s correlation was performed and established a very strong relationship (ρ > 0.9) in most cases for the information associated with the *C. brachyurus* and *L. guttulus* samples and a moderate relationship (0.5 ≤ ρ ≥ 0.7) for the *P. yagouaroundi* samples. The other samples in this group did not show significant degrees of correlation (ρ < 0.5). After associating the three identification techniques, a final classification of host species was conducted using the highlighted information (Table 1).

### Parasitological diagnosis

In the faeces of the animals, several parasitic taxa were identified, including the phyla Nematoda, Platyhelminthes and Protozoa. These were characterized into morphotypes by their different sizes, colours and shapes. Out of the 244 faecal samples collected, structures of gastrointestinal parasites were revealed in 198 (81.1%) through the combined use of microscopic coproparasitological techniques and ELISA. In general, helminths were observed more frequently than protozoa and were mainly seen in faeces from carnivores. The inverse was observed in relation to the stool samples from artiodactyls (Table 2).

**Table 2:** Frequency of gastrointestinal parasites in carnivorous and artiodactyls faecal samples surveyed in Itatiaia National Park, Brazil

Helminth and protozoan structures	Order Carnivora (*n* = 213)	Order Artiodactyla (*n* = 31)	Total (*n* = 244)
**Helminths**			
Family Ascarididae	71 (33.3%)	4 (12.9%)	75 (30.8%)
*Trichuris* sp.	31 (14.5%)	–	31 (12.7%)
*Capillaria* sp.	29 (13.6%)	–	29 (11.9%)
Nematode larvae	25 (11.7%)	6 (19.4%)	31 (12.7%)
Thin-shelled nematode egg	21 (9.8%)	3 (9.7%)	24 (9.8%)
*Physaloptera* sp.	12 (5.6%)	–	12 (4.9%)
Family Diphyllobothriidae	52 (24.1%)	–	52 (21.3%)
Order Cyclophyllidea	8 (3.7%)	–	8 (3.3%)
Family Dicrocoeliidae	10 (4.7%)	–	10 (4.1%)
Phylum Acanthocephala	1 (0.5%)	–	1 (0.4%)
Subtotal of helminths positive samples	151 (70.9%)	10 (32.2%)	161 (66%)
**Protozoan**			
Non-sporulated coccidian	10 (4.7%)	–	10 (4.1%)
*Eimeria* sp.	–	1 (3.2%)	1 (0.4%)
*Balantioides coli*	–	6 (19.4%)	6 (2.4%)
Amoebae	3 (1.4%)	–	3 (1.2%)
Coproantigens of *Cryptosporidium* sp.	42 (19.7%)	25 (80.6%)	67 (27.4%)
Subtotal of protozoan positive samples	54 (25.3%)	27 (87.1%)	81 (33.2%)
**Total of positive samples**	**171 (80.3%)**	**27 (87.1%)**	**198 (81.1%)**

Among the 213 faecal samples from animals of the order Carnivora that were analysed, 171 (80.3%) showed structures from gastrointestinal parasites, among which eggs of the families Ascarididae and Diphyllobothriidae and coproantigens of *Cryptosporidium* sp. can be highlighted. Among the samples from the animals of the order Artiodactyla, the total number positive for gastrointestinal parasites was 27 samples (87.1%). Antigens of *Cryptosporidium* sp. were the most frequently detected structure, followed by cysts of *Balantioides coli* and nematode larvae (Table 2).

**Fig. 1:**
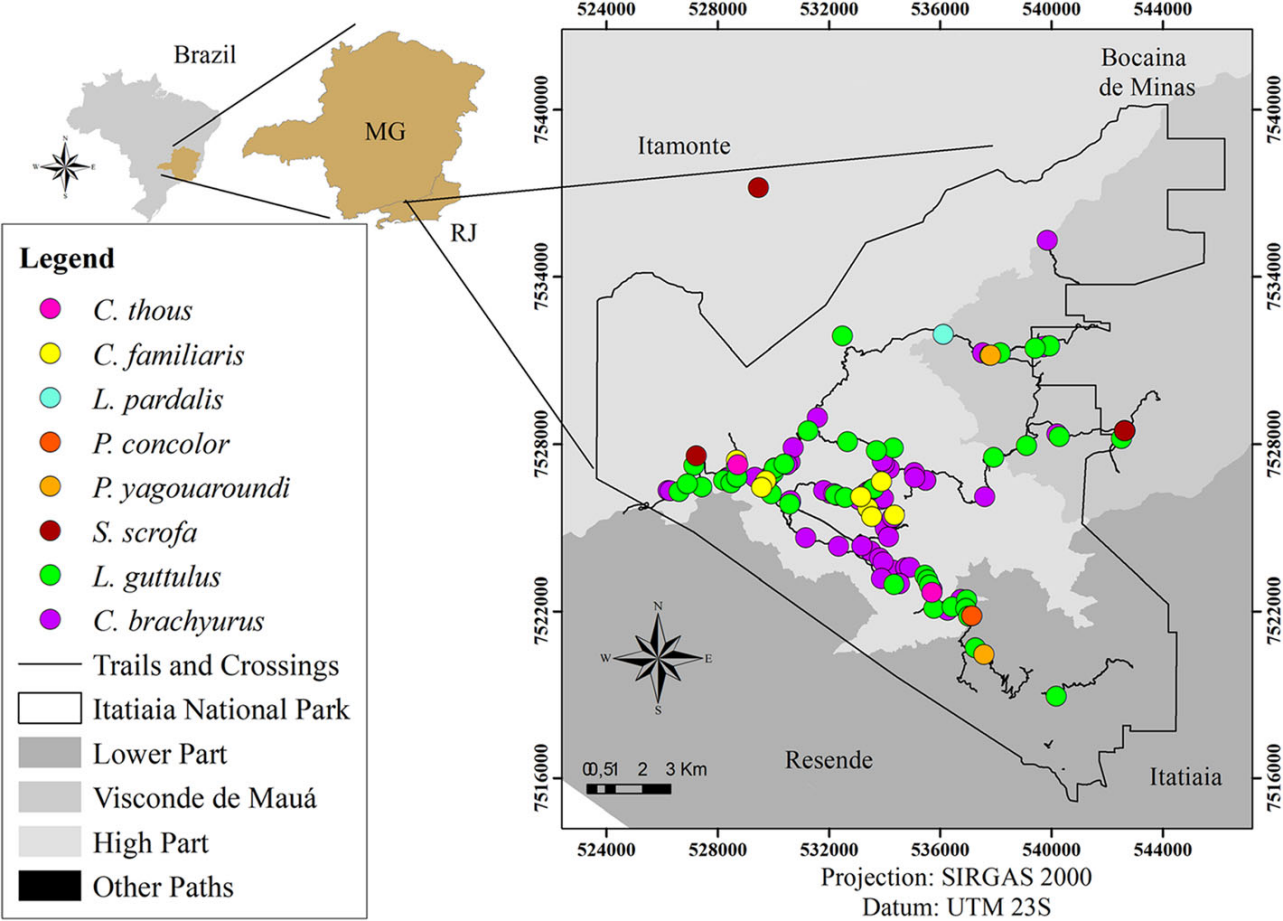
Collection points for faecal samples positive for parasites in Itatiaia National Park. (Arcgis version 10.5)

**Fig. 2:**
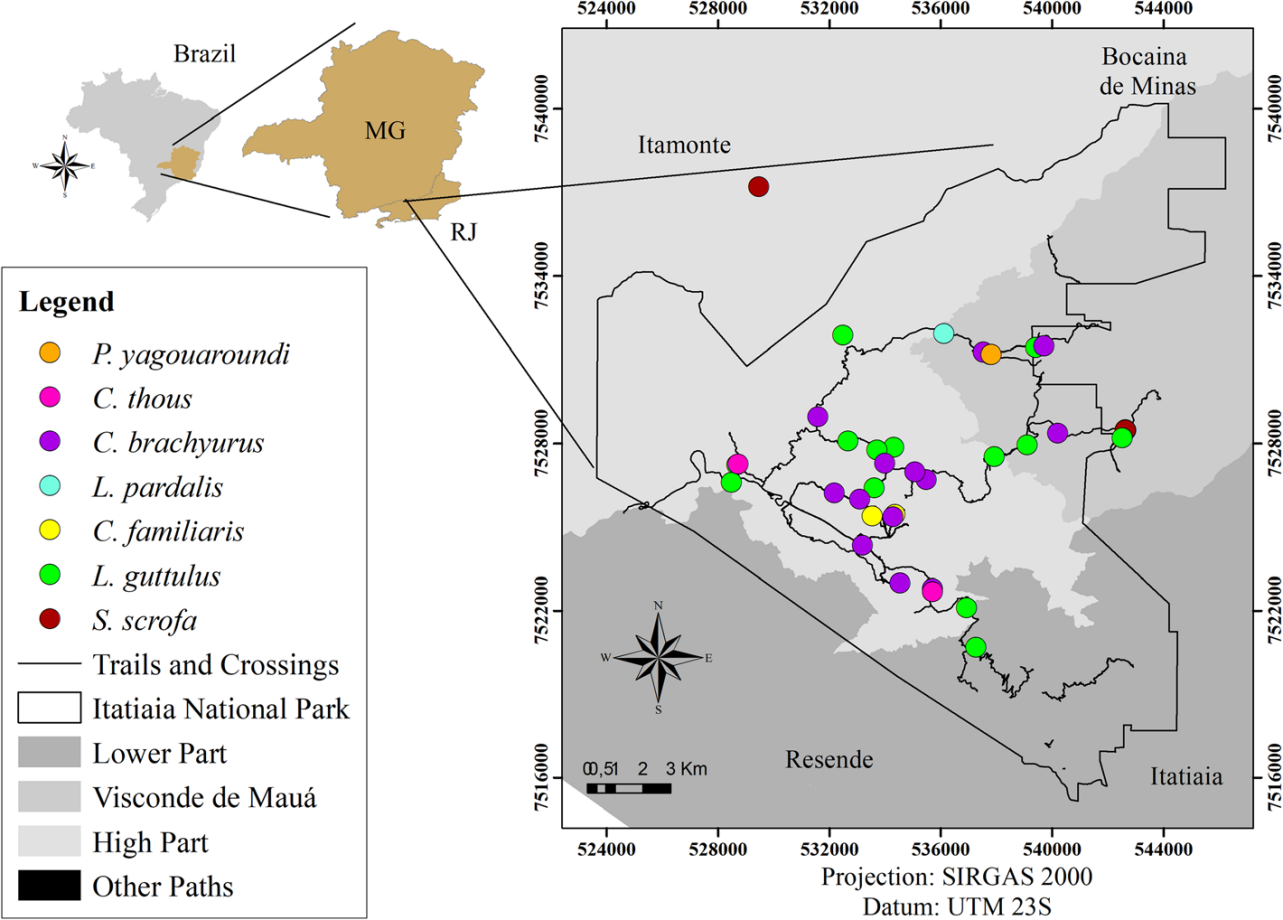
Collection points for faecal samples from carnivores and an artiodactyl positive for Cryptosporidium sp. (Arcgis version 10.5)

Out of the 244 faecal samples retrieved from the park, most were identified as belonging to *C. brachyurus* (97) and *L. guttulus* (52). Among these, structures of gastrointestinal parasites were detected in 79 (81.4%) samples from *C. brachyurus* and in 43 (82.7%) from *L. guttulus* (Figs. 1 and 2). Such parasites were also observed in other faecal samples of carnivores and artiodactyls that had been collected along the park trails (Figs. 1 and 2).

**Fig. 3:**
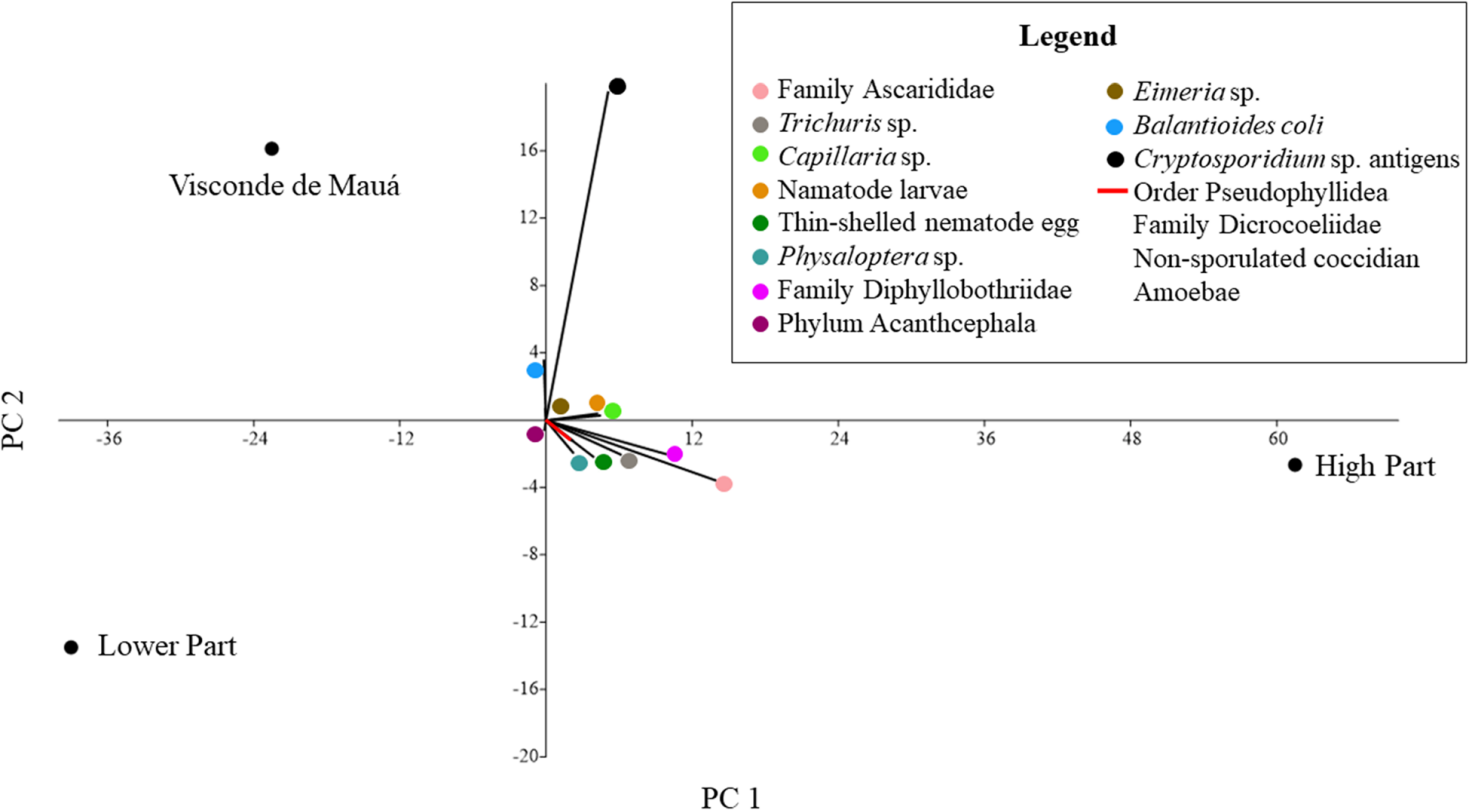
Distribution of parasitic taxa detected in fecal samples of carnivores and artiodactyl in the three parts of the park: High Part, Lower Part and Visconde de Mauá through Principal component analysis (Past version 3.2.2)

In addition, a difference in the distribution of parasitic structures can be observed in the three areas of the park. The High Part was the region where all parasitic taxa were detected, except for *Eimeria* sp. However, the Lower Part was the region that presented the least diversity of parasitic structures, being detected 7 out of 15 taxa. In Visconde de Mauá, 10 out of 15 taxa were detected, highlighting the presence of antigens of *Cryptosporidium* sp. which was quite evident (Fig. 3).

**Fig. 4:**
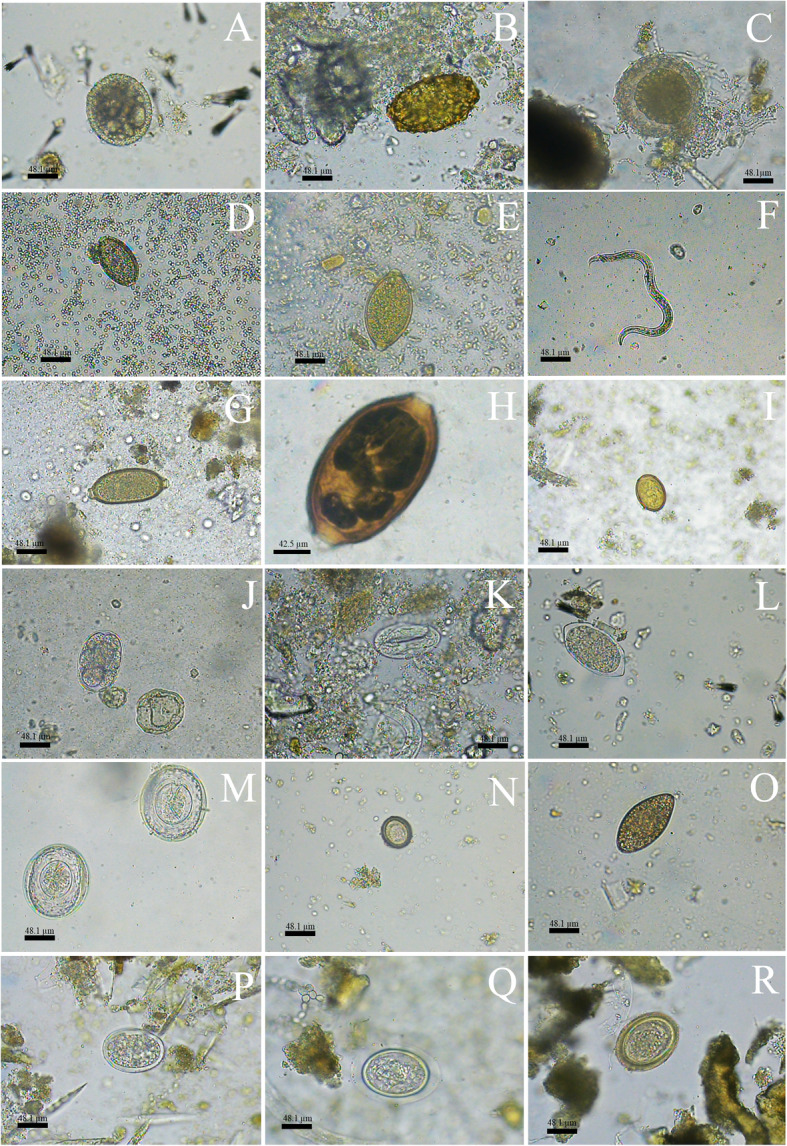
Morphotypes of helminths eggs in 400 x (A to F; H to S) and 1000 x (G) detected in faecal samples of carnivores and artiodactyls form Itatiaia National Park, Brazil. **a** Ascarididae family 1. **b** Ascarididae family 2. **c** Ascarididae family 3. **d** *Trichuris* sp. 1. **e** *Trichuris* sp. 2. **f** Nematode larvae. **g** *Capillaria* sp. 1. **h** *Capillaria* sp. 2. **i** Dicrocoellidae family. **j** Thin-shelled nematode egg 1. **k** Thin-shelled nematode egg 2. **l** Thin-shelled nematode egg 3. **m** Cyclophyllidea order 1. **n** Cyclophyllidea order 2. **o** Diphyllobothriidae family. **p** *Physaloptera* sp. **q** Acanthocephala phylum 1. **r** Acanthocephala phylum 2

Eggs of the family Ascarididae, especially those classified as morphotype 1, that were similar to *Toxocara* sp. were detected mainly in faecal samples from carnivores, except from *L. pardalis* and *P. concolor*. Parasite structures that matched the typical morphology of infertile *Ascaris* eggs (morphotype 2) were only observed in samples from *S. scrofa*. A third morphotype of ascarid was detected in 11.9% of the samples, including the faeces from *C. brachyurus*, *C. familiaris* and *L. guttulus* (Table 3 and Fig. 4).

**Table 3:** Frequency of gastrointestinal parasite morphotypes in mammal faecal samples from Itatiaia National Park, Brazil

Morphotypes of helminth and protozoan structures	*Chrysocyon brachyurus* (*n* = 97)	*Canis familiaris* (*n* = 13)	*Cerdocyon thous* (*n* = 2)	*Leopardus guttulus* (*n* = 52)	*Puma yagouaroundi* (*n* = 2)	*Leopardus pardalis* (*n* = 1)	*Puma concolor* (*n* = 1)	Order Carnivora US (*n* = 45)	*Sus* *scrofa* (*n* = 12)	Order Artiodactyla US (*n* = 19)	Total (*n* = 244)
**Helminths**											
Family Ascarididae	25 (25.8%)	4 (30.8%)	1 (50%)	20 (38.5%)	1 (50%)	–	–	21 (46.7%)	4 (33.3%)	–	75 (30.8%)
Morphotype 1 - **C**: 92.5 ± 5.8 × 81.4 × 7.1; **F**: 92.5 ± 6 × 81.4 × 6.2; S: 88.8 ± 8.7 × 85.1 × 7.6	20 (20.6%)	2 (15.4%)	1 (50%)	19 (36.5%)	1 (50%)	–	–	15 (33.3%)	3 (25%)	–	61 (25%)
Morphotype 2 - **C**: 111 ± 18.5 × 107.3 ± 23.2; **F**: 107.3 ± 8.9 × 83.2 ± 12.9	–	–	–	–	–	–	–	–	1 (8.3%)	–	1 (0.4%)
Morphotype 3 - **S**: 114.7 ± 4.8 × 74 ± 1.6	11 (11.3%)	3 (23.1%)	–	10 (19.2%)	–	–	–	4 (8.9%)	–	–	29 (11.9%)
*Trichuris* sp.	19 (19.6%)	–	–	4 (7.7%)	2 (100%)	–	–	6 (13.3%)	–	–	31 (12.7%)
Morphotype 1 - **C**: 88.8 ± 8 × 37 ± 2.8; **F**: 81.4 ± 4.5 × 40.7 ± 4.2	12 (12.4%)	–	–	1 (1.9%)	2 (100%)	–	–	5 (11.1%)	–	–	18 (7.4%)
Morphotype 2 - **C**: 107.3 ± 7.3 × 40.7 ± 4.9; **F**: 107.3 ± 7.6 × 42.5 ± 3.7	10 (10.3%)	–	–	4 (7.7%)	–	–	–	2 (4.4%)	–	–	18 (7.4%)
*Capillaria* sp.	14 (14.4%)	–	–	11 (21.1%)	1 (50%)	1 (100%)	–	2 (4.4%)	–	–	29 (11.9%)
Morphotype 1 - **C**: 85.1 ± 5.1 × 44.4 ± 4.9; **F**: 85.1 ± 6.4 × 44.4 ± 3.9	8 (8.2%)	–	–	6 (11.5%)	1 (50%)	1 (100%)	–	–	–	–	15 (6.1%)
Morphotype 2 - **C**:99.9 ± 8.1 × 44.4 ± 6.1; **F**: 101.7 ± 5.6 × 51.8 ± 6.5	9 (9.3%)	–	–	10 (19.2%)	–	1 (100%)	–	2 (4.4%)	–	–	23 (9.4%)
Nematode larvae	13 (13.4%)	–	–	5 (9.6%)	–	–	1 (100%)	6 (13.3%)	1 (8.3%)	5 (26.3%)	31 (12.7%)
Thin-shelled nematode egg	9 (9.3%)	3 (23.1%)	1 (50%)	4 (7.7%)	–	–	–	4 (8.9%)	2 (16.7%)	1 (5.3%)	24 (9.8%)
Morphotype 1 - **C**: 85.1 ± 4.2 × 55.5 ± 3.7; **F**: 96.2 × 40.7	7 (7.2%)	1 (7.7%)	–	1 (1.9%)	–	–	–	3 (6.7%)	–	–	12 (4.9%)
Morphotype 2 - **C**: 107.3 ± 5.5 × 57.3 ± 4.8; **F**: 109.1 ± 2.6 × 85.1 ± 31.4; **S**: 172 ± 29.8 × 79.5 ± 10.6	2 (2.1%)	1 (7.7%)	1 (50%)	3 (5.8%)	–	–	–	1 (2.2%)	2 (16.7%)	–	10 (4.1%)
Morphotype 3 - **C**: 92.5 ± 6.1 × 55.5 ± 5.6	–	1 (7.7%)	–	–	–	–	–	–	–	1 (5.3%)	2 (0.8%)
*Physaloptera sp.-* **C**: 70.3 ± 10 × 46.2 ± 9.7; **F**: 70.3 × 51.8	9 (9.3%)	–	–	1 (1.9%)	–	–	–	2 (4.4%)	–	–	12 (4.9%)
Family Diphyllobothriidae - **C**:88.8 ± 9.3 × 48.1 ± 6.3; **F**: 85.1 ± 7.1 × 48.1 ± 4	31 (32%)	–	1 (50%)	10 (19.2%)	–	–	–	10 (22.2%)	–	–	52 (21.3%)
Order Cyclophyllidea	4 (4.1%)	1 (7.7%)	–	1 (1.9%)	–	–	–	2 (4.4%)	–	–	8 (3.3%)
Morphotype 1 - **C**: 83.2 ± 8.1 × 77.7 ± 12.3; **F**: 81.4 ± 15.7 × 75.8 ± 7.8	3 (3.1%)	1 (7.7%)	–	1 (1.9%)	–	–	–	2 (4.4%)	–	–	7 (2.9%)
Morphotype 2 - **C**: 48.1 × 37	1 (1%)	–	–	–	–	–	–	–	–	–	1 (0.4%)
Family Dicrocoeliidae - **C**: 55.5 ± 11.3 × 33.3 ± 3.7; **F**: 51.8 ± 1.5 × 33.3 ± 3.3	5 (5.1%)	–	–	3 (5.8%)	–	–	–	2 (4.4%)	–	–	10 (4.1%)
Phylum Acanthocephala	–	–	–	–	–	–	–	1 (2.2%)	–	–	1 (0.4%)
Morphotype 1–88.8 ± 9.5 × 74 ± 3.3	–	–	–	–	–	–	–	1 (2.2%)	–	–	1 (0.4%)
Morphotype 2–85.1 × 66.7	–	–	–	–	–	–	–	1 (2.2%)	–	–	1 (0.4%)
Helminths positive samples	72 (74.2%)	7 (53.8%)	1 (50%)	35 (67.3%)	2 (100%)	1 (100%)	1 (100%)	31 (68.9%)	5 (41.7%)	5 (26.3%)	161 (66%)
**Protozoa**											
Non-sporulated coccidian	9 (9.3%)	–	–	–	–	–	–	1 (2.2%)	–	–	10 (4.1%)
Morphotype 1 - **C**: 25.9 ± 2.4 × 22.2 ± 2.5	7 (7.2%)	–	–	–	–	–	–	–	–	–	7 (2.9%)
Morphotype 2 - **C**: 37 ± 6.9 × 33.3 ± 5.6	5 (5.1%)	–	–	–	–	–	–	–	–	–	5 (2%)
*Eimeria* sp. - **S:** 37 × 37	–	–	–	–	–	–	–	–	1 (8.3%)	–	1 (0.4%)
*Balantioides coli -* **S**: 48.1 ± 38.8 × 44.4 ± 35.5	–	–	–	–	–	–	–	–	2 (16.7%)	4 (21%)	6 (2.4%)
Amoebae - **C**: 18.5 ± 5.8 × 18.5 ± 3.6; **F**: 18.5 ± 3.2 × 18.5 ± 2	2 (2.1%)	–	–	1 (1.9%)	–	–	–	–	–	–	3 (1.2%)
*Cryptosporidium* sp. coproantigens	13 (13.4%)	2 (15.4%)	2 (100%)	14 (26.9%)	1 (50%)	1 (100%)	–	9 (20%)	10 (83.3%)	15 (78.9%)	67 (27.4%)
Protozoan positive samples	22 (22.7%)	2 (15.4%)	2 (100%)	15 (28.8%)	1 (50%)	1 (100%)	–	10 (22.2%)	10 (83.3%)	17 (89.5%)	81 (33.2%)
**Total positive samples**	79 (81.4%)	8 (61.5%)	2 (100%)	43 (82.7%)	2 (200%)	1 (100%)	1 (100%)	35 (77.8%)	10 (83.3%)	17 (89.5%)	198 (81.1%)

Eggs of the family Diphyllobothriidae were the second most frequent parasite among the helminths and were detected only in the faecal samples from carnivores. Nematode larvae and *Trichuris* sp. eggs were observed in 12.7% of the stool samples. Nematode larvae were detected in faeces from both carnivores and artiodactyls and were not classified into different morphotypes. Eggs of *Trichuris* sp. were only diagnosed in samples from carnivores, and these were morphologically classified as morphotypes 1 and 2. *Capillaria* sp. eggs were diagnosed in 11.9% of the faecal samples analysed. These were classified into morphotypes 1 and 2 and were detected in faeces from *C. brachyurus, L. guttulus, P. yagouaroundi* and *L. pardalis* (Table 3 and Fig. 4).

Thin-shelled nematode eggs were observed in 9.8% of the samples. Among these, the eggs were classified into morphotype 1, which were similar to those of the superfamily Strongyloidea, and morphotype 2, which were similar to strongylids (superfamilies Trichostrongyloidea and Strongyloidea). Morphotype 1 eggs were detected in faeces from *C. brachyurus*, *C. familiaris* and *L. guttulus,* and morphotype 2 eggs were detected in samples from *L. guttulus*, *C. thous* and *S. scrofa*. Thin-shelled eggs with tapered ends were diagnosed in the faeces of both *C. familiaris* and unidentified artiodactyls (Table 3 and Fig. 4).

In the faeces from carnivores, eggs of other helminths, such as *Physaloptera* sp. and the family Dicrocoeliidae were also observed. Cestode eggs of the order Cyclophyllidea, which were classified as morphotype 1, were detected in 2.9% of the faeces analysed. Eggs from the family Taeniidae, which were named morphotype 2, were detected in one sample from *C. brachyurus*. In a faecal sample that was positive for eggs of the phylum Acanthocephala, the host was only characterized down to the taxonomic group of the order Carnivora (Table 3 and Fig. 4).

**Fig. 5:**
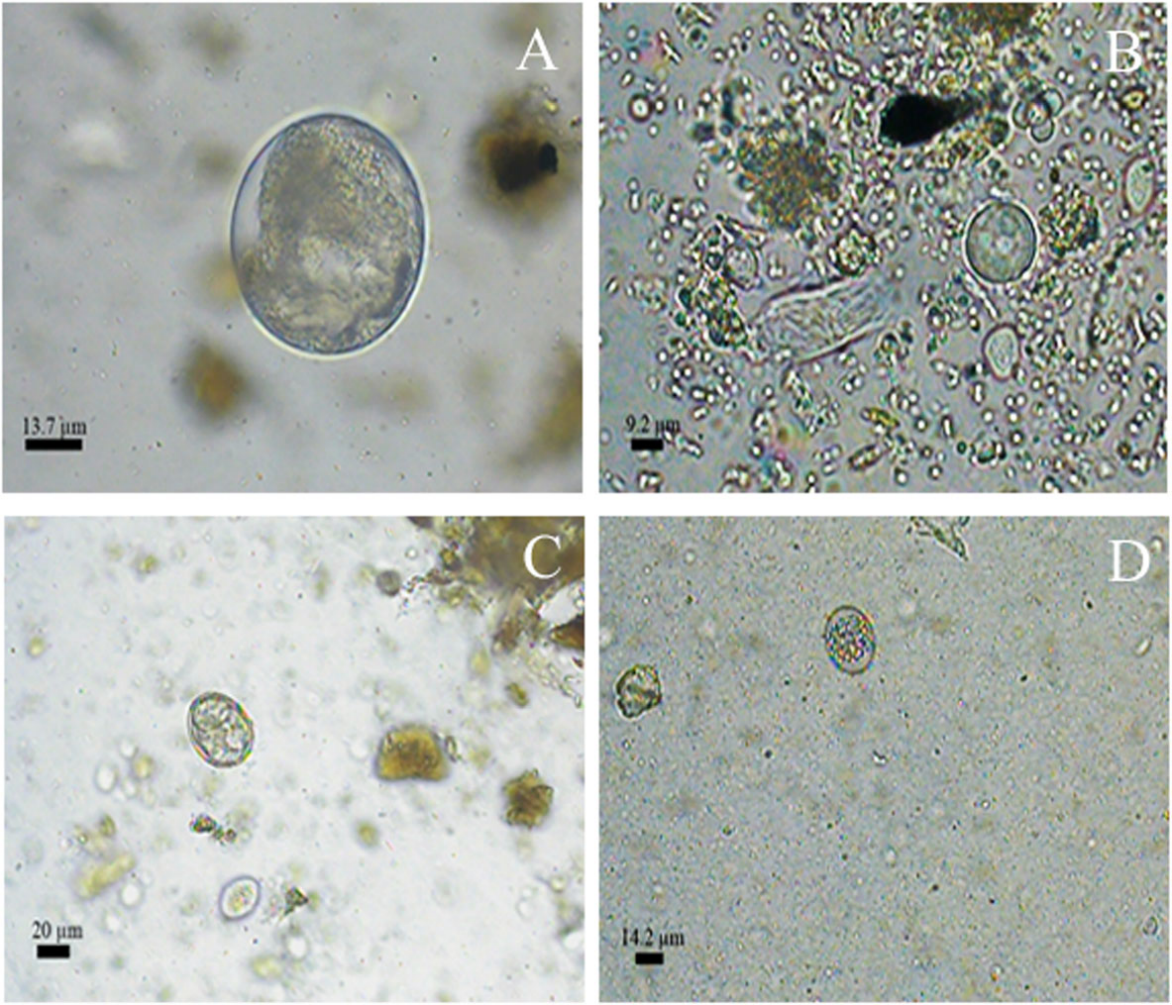
Morphotypes of protozoa cysts and oocysts in 1000 x detected in faecal samples of carnivores and artiodactyls form Itatiaia National Park, Brazil. **a** *Balantioides coli*. **b** Amoebae. **c** *Eimeria* sp. **d** Non-sporulated coccidia

Among the protozoa detected, *Cryptosporidium* sp. was diagnosed through antigens in the faeces of all animals that were identified to the species level except for *P. concolor* (Fig. 2). Unsporulated coccidia oocysts and tetranucleated amoeba cysts were detected in faeces that were identified as from *C. brachyurus,* and the latter were also found in faeces from *L. guttulus*. Sporulated coccidian oocysts with the typical morphological pattern of *Eimeria* sp. and *Balantioides coli* cysts were detected only in faeces from artiodactyls (Table 3 and Fig. 5). All different morphotypes of protozoa structures detected presented similar morphology and varied only in size.

Regarding parasitic associations, polyparasitism was observed in 94 samples (38.5%), which had two to eight parasite structures. Associations were found between helminths alone, between helminths and protozoa and between protozoa alone. The most frequently detected parasitic associations occurred between eggs of the families Ascarididae and Diphyllobothriidae and between eggs of Ascarididae and *Trichuris* sp.; both combinations were present in five samples from carnivores (2.3%). In the faeces from artiodactyls, the most frequent association occurred between nematode larvae and *Cryptosporidium* sp. coproantigens in three samples (9.7%).

**Fig. 6:**
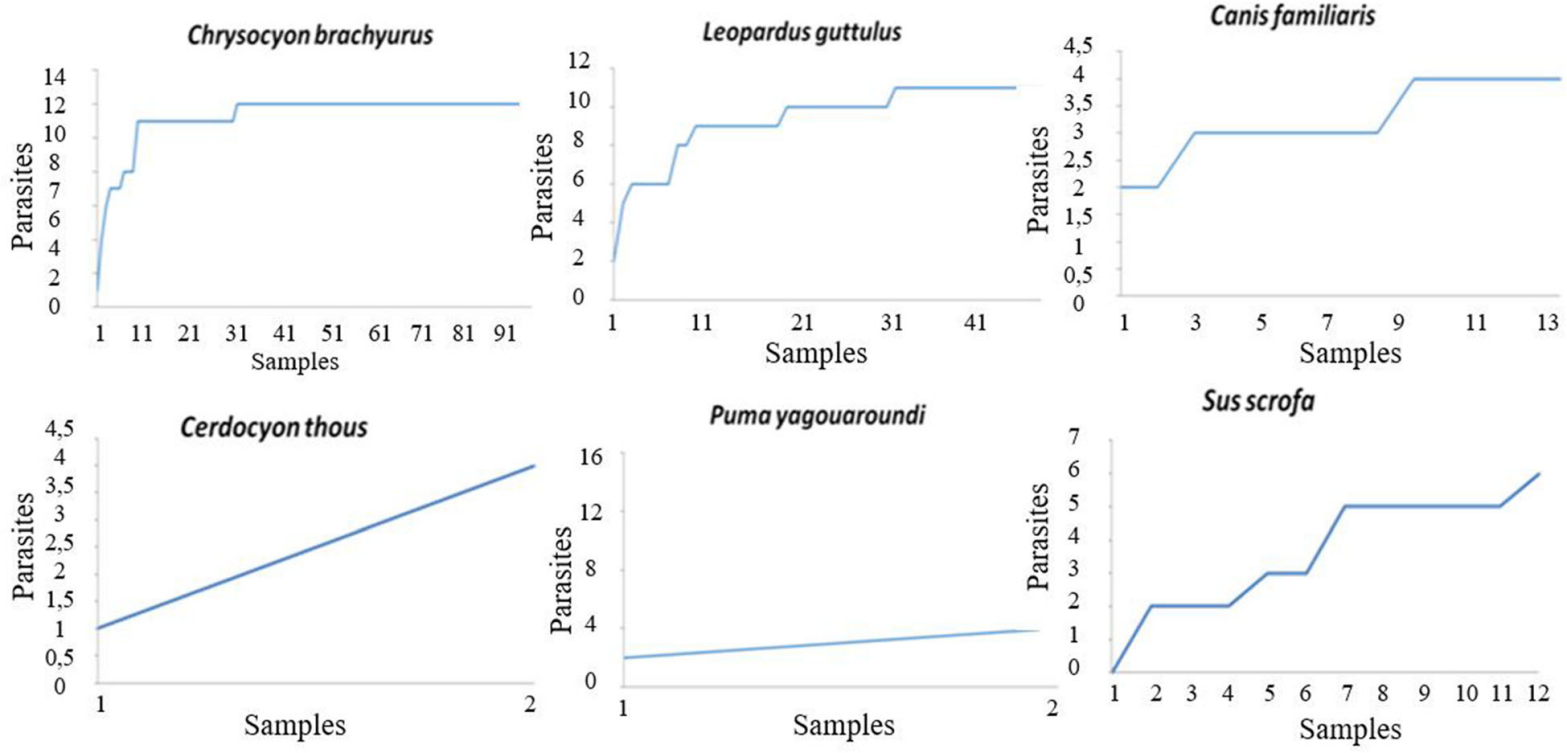
Accumulation curves for gastrointestinal parasite structures detected in faecal samples from carnivores and an artiodactyl from Itatiaia National Park, Brazil

To analyse the richness, diversity, and similarity indices, the sample sufficiency for each host species (relationship between the faecal samples and the different parasitic taxa detected) was analysed and plotted on accumulation curves. The accumulation curves of *C. brachyurus*, *L. guttulus*, and *C. familiaris* stabilized, which means that the number of samples recovered for each of these hosts was enough to estimate the richness and diversity indices of the parasitic fauna. It was not possible to establish accumulation curves for *L. pardalis* and *P. concolor* since only one faecal sample was collected from each of these species (Fig. 6).

**Fig. 7:**
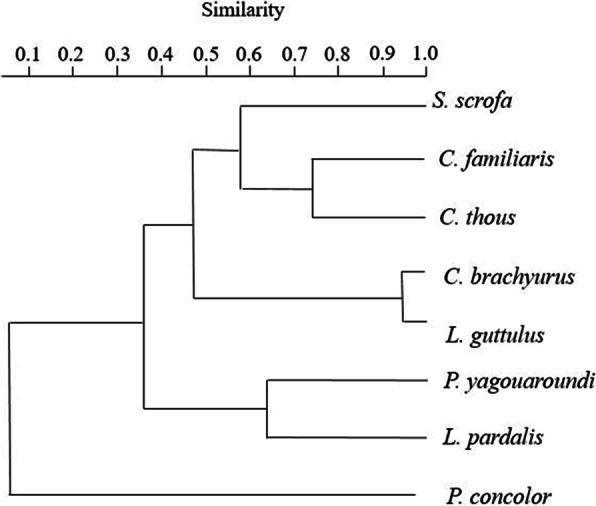
Dendrogram generated from cluster analysis (UPGMA) using the Sorensen similarity index for comparison of parasite structures detected in faecal samples from carnivores and an artiodactyl collected in Itatiaia National Park, Brazil

The faecal samples from *C. brachyurus* and *L. guttulus* presented the highest richness and were positive for a great number of different parasite taxa, and similarity, as demonstrated by the parasitic likeness between the hosts (Table 4 and Fig. 7). However, despite the detection of common parasites between artiodactyls and carnivores, many agents were detected in only one of these hosts, which justified the low parasite similarity index between them (Fig. 7). In the pooled t test, the Shannon diversity index (H′) of the parasites was significant (*p* < 0.05) between *C. brachyurus* and *C. familiaris* and between *S. scrofa* and *C. brachyurus* (Table 5). Thus, it was demonstrated that the parasitic taxa and their distribution differed considerably among hosts.

**Table 4:** Richness and diversity of gastrointestinal parasites in mammal faeces from Itatiaia National Park, Brazil

Host	Richness	Shannon (H′)	Simpson
Family Canidae			
*Chrysocyon brachyurus*	12	2.2761	0.887
*Canis familiaris*	4	1.2799	0.778
*Cerdocyon thous*	4	1.3297	0.867
Family Felidae			
*Leopardus guttulus*	11	1.9662	0.850
*Puma yagouaroundi*	4	1.3322	0.900
*Leopardus pardalis*	2	0.6931	1
*Puma concolor*	2	0.6931	1
Family Suidae			
*Sus scrofa*	6	1.4286	0.721

**Table 5:** Statistical significance of Poole t test of identified hosts from Itatiaia National Park, Brazil

pvalue	*Leopardus guttulus*	*Chrysocyon brachyurus*	*Canis familiaris*	*Cerdocyon thous*	*Leopardus. pardalis*	*Sus scrofa*	*Puma yagouaroundi*	*Puma concolor*
*Leopardus guttulus*		0.05	0.12	0.34	0.46	0.1	0.43	0.46
*Chrysocyon brachyurus*			0.03*	0.17	0.38	0.01*	0.26	0.38
*Canis familiaris*				0.94	0.72	0.76	0.95	0.72
*Cerdocyon thous*					0.7	0.88	1	0.7
*Leopardus pardalis*						0.66	0.7	1
*Sus scrofa*							0.9	0.66
*Puma yagouaroundi*								0.6
*Puma concolor*								

*p value <0.05

## Discussion

From the association of the macroscopic, trichological and DNA sequencing results, a final identification of the host species was obtained. Through macroscopic examination of the faeces, problems in identification occurred with canid samples when seeds, which are common in the diet of neotropical canids such as *C. brachyurus*, were not observed. In addition, there is no consensus among authors regarding the shape of mammalian stool. Thus, to minimize misidentification of the host by macroscopy, the samples were classified only into high-level taxonomic categories, such as order, in most cases. Despite the low resolution of host identification, this is a low-cost technique and can be used by any researcher during field work.

In this study, host species were not identified through trichological analysis in most of the samples. As pointed out at Serra dos Órgãos National Park, one of the limitations in mammalian identification through trichology is that faeces often presents many hairs, but not all are guard hairs [16]. In addition, some guard hair had deteriorated, making it impossible to assess their cuticles. In this case, since only the medullary patterns were observed, it was only possible to identify the animals down to the family level, such as the family Canidae. On the other hand, the guard hairs recovered from artiodactyl samples did not have cuticles, and only the internal layer was composed of fibres. The loss of the cuticular layer may have occurred during laboratory processing or by the passage of the guard hairs through the gastrointestinal tract. Nevertheless, the cuticle may have been rubbed off when artiodactyls rubbed their bodies on natural substrates, such as trees. It is noteworthy that trichological processing is time-consuming, and the assessment of guard hairs requires trained professionals. Moreover, it is important to make controls with guard hairs that were taken directly from animals to achieve greater accuracy in host identification from the non-invasive samples. Nevertheless, this methodology is also very advantageous since it is inexpensive and allows a very specific taxonomic classification of the animal.

DNA sequencing was also used for host identification. It is important to highlight that genetic sequences do not ensure that it is the true host due to the possibility of countermarking by other animals with territorial behaviour. Nonetheless, the use of DNA sequencing may be the only alternative for the identification of *S. scrofa*, for which faecal hair release in feces is not common. However, this technique was the most expensive among those used in this study, and it does not always provide satisfactory results due to many faecal inhibitors. Thus, the association among macroscopy, trichology, and DNA sequencing is essential to provide the most trustworthy identification from non-invasive samples.

The overall positive rate for gastrointestinal parasites was 81.1%. In this study, from all the 244 samples, the positive rates were 36.5% for Canidae family, 19.3% for Felidae and 4.1 for Suidae. Lower overall frequencies have been reported in other studies, varying from 58 to 74.7% for feline faeces from reserves and forests in Mexico [18–20], 75% for feline and artiodactyl faeces from a reserve in Bolivia [21], 53.3% for faeces from canids, felids, mustelids and procyonids collected in a reserve in Minas Gerais, Brazil [22], and 70% for feline faeces found in a reserve in Espírito Santo, Brazil [23]. In contrast, the frequency of parasites detected in faeces from Serra dos Órgãos National Park was 86.6%, which was slightly higher than that observed in Itatiaia National Park [16]. The high frequency of gastrointestinal parasites detected may have occurred since these animals live in natural environments that are rich in abiotic and biotic factors that promote infection through contact with contaminated soil, water, food and infected prey.

In general, helminths were more frequent than protozoa in the faeces from carnivores. High frequencies of helminths have also been observed in stool samples from carnivores in Thailand, Mexico, Bolivia and Brazil [16, 18–29]. In this study, the high frequency of helminths showed that the environment inhabited by the animals presented conditions that were favourable for maintenance of nematode, cestode, trematode and acanthocephalan life cycles. Unlike in carnivore faecal samples, protozoa were the most frequently detected parasites in the faeces of *S. scrofa*. Among the studies that analysed samples of free-living *S. scrofa*, *Tayassu pecari* and *Pecari tajacu*, only one conducted in Texas, USA, reported protozoa in faeces from pigs [30].

Eggs from the family Ascarididae were the most frequently observed group in the faecal material of carnivorous hosts. Most of the eggs detected were similar to *Toxocara* sp. (morphotype 1) and were consistent with *T. canis* and *T. cati*. Brownish ascarid eggs with very thick shells (morphotype 3) were also detected. These eggs may have belonged to another species that has not yet been reported or may even have resulted from parasitic adaptation. In Itatiaia, the high positivity for *Toxocara* sp. may have been favoured due to the extreme resistance of these ascarid eggs the park’s abundant edaphic environment and humid tropical climate, which contribute towards larval development within the egg. Moreover, predation can be cited as a possible form of transmission due to paratenic hosts [24] and pseudoparasitism, in which the animal does not truly become infected.

Eggs of the family Diphyllobothriidae were the second most frequently detected parasite structures among helminths in faeces from carnivores. Morphologically, the eggs were consistent with *Spirometra* sp. [31]. The high frequency of these parasites seemed to be related to the abundance of rivers and waterfalls in the park, where copepods, fish, amphibians and snakes (intermediate hosts) can be found.

Nematode larvae were detected in the faeces of *C. brachyurus*, *L. guttulus*, *P. concolor* and *S. scrofa*. These species have also been reported in faeces from felids at a reserve in São Paulo and in the Serra dos Órgãos National Park in Rio de Janeiro, both of which are in Brazil [16]. Although no specific classification of the larvae was made, it was verified if the larvae detected belonged to the genus *Aelurostrongylus* and none presented a subterminal spine structure. Since the faecal samples were collected directly from the ground, the larvae observed may have been free-living nematodes. In addition, these larvae could have developed from pathogenic parasites such as hookworm eggs, *Strongyloides* eggs or even, in the case of artiodactyls, strongylid eggs if the samples were collected from the rectum of the animals or immediately after the they defecated.

Thin-shelled nematode eggs were observed in faecal material from both the carnivores and the artiodactyls and appeared similar to hookworm eggs (morphotype 1). Strongylid-like eggs (morphotype 2) were specifically detected in artiodactyl faeces. This finding was expected and corroborated the host identification. Strongylid eggs have been reported in faeces of artiodactyls in a reserve in the Pantanal and in a park in Piauí, Brazil [25, 32]. It is important to highlight that strongylid-like eggs were also diagnosed in faeces from carnivores. This may have occurred due to ungulate or wild artiodactyl predation, farms that had not yet been removed from the park, or ingestion of contaminated water or food. It is important to highlight that those remained farms in PNI can be the source for the introduction of new parasites among the wild fauna that can disseminate the eggs in the park causing an environmental imbalance in the future.

Eggs of *Trichuris* sp. and *Capillaria* sp. were exclusively detected in faeces from carnivores. The *Trichuris* sp. morphotype 1 eggs detected in the *C. brachyurus* samples were consistent with *Trichuris vulpis,* and those diagnosed in the faeces of *L. guttulus* were similar to *Trichuris campanula*. The morphotype 1 and 2 eggs of *Capillaria* sp. and morphotype 2 eggs of *Trichuris* sp. were much larger than the eggs already described as infecting canids and felids in the literature. This demonstrated that other enoplids were probably infecting these animals. It should also be pointed out that these species of *Trichuris* and *Capillaria* may have been ingested during the predation of infected rodents and represent cases of pseudoparasitism. Overall, the diagnosis of *Capillaria* sp. in the biological material of wild animals seems to be directly related to predation on rodents [33]. This may have occurred in the present study, since rodent guard hairs were observed in most of the faecal samples that were positive for *Capillaria* sp.

Eggs of other helminths were also detected in fecal samples from the park, such as the nematode *Physaloptera* sp., dicrocoelids similar to *Platynosomum illiciens*, cestodes of the order Cyclophyllidea and acanthocephalans.

In general, the biological life cycles of these parasites require the existence of different intermediate and paratenic hosts. In Itatiaia National Park, the animals that can be intermediate and paratenic hosts for these parasites are very prevalent, especially arthropods, rodents and reptiles, thus demonstrating that this is an appropriate environment for the maintenance of heteroxenic life cycles in which different host species participate.

Among the protozoa, the phylum Apicomplexa was most frequently diagnosed in Itatiaia National Park, including non-sporulated coccidian oocysts in faeces from *C. brachyurus*. Sporulated coccidian oocysts of *Eimeria* sp. (which were detected in faeces from *S. scrofa*) and coproantigens of *Cryptosporidium* sp. were proportionally more frequently detected in the faeces from artiodactyls.

The unsporulated coccidian oocysts that were detected in faecal material from *C. brachyurus* presented different sizes. The smallest was morphotype 1, which was consistent with *Cystoisospora ohioensis,* and the largest was morphotype 2, which was similar to *Cystoisospora canis* [34]. *Cystoisospora* is the most commonly reported genus in the coccidian group in faeces from wild felids in parks and reserves in Mexico and Brazil [16, 20, 22, 35].

The use of an immunoenzymatic assay for the detection of *Cryptosporidium* sp. may have facilitated its diagnosis in the present study due to the greater sensitivity of this technique. It is noteworthy that the artiodactyl diet, i.e., intake of forage, seemed to be a factor that determined a higher frequency of *Cryptosporidium* sp. in this group. It is important to highlight that approximately 31 species of *Cryptosporidium* sp. have been described, among which some have high zoonotic potential to infect mammals [36–39].

The high positivity for *Cryptosporidium* sp. in the carnivores and artiodactyls of Itatiaia National Park is extremely important, since there are very few reports of parasitism in these animals, thus emphasizing the need for further studies to be conducted in wild environments. In addition, it is important to draw attention to the possibility that this protozoon is being introduced to the park by invasive artiodactyls (e.g., *S. scrofa*). It needs to be highlighted that the identification of *Cryptosporidium* sp. in samples of an introduced species should be regarded as a warning, since there are no reports in the Brazilian literature of these parasites infecting these animals.

The identification of protozoan oocysts and coproantigens in faeces from carnivores and artiodactyls is directly associated with the ingestion of sporulated oocysts, mainly through water consumption or through predation. In the case of *Cystoisospora* sp., carnivores and artiodactyls become infected by ingesting cysts containing zoites within the tissues of intermediate hosts, such as mammals, birds and rodents. Both forms of infection may be occurring in Itatiaia National Park given the abundance of possible prey for these hosts, as well as the richness of water resources, such as lakes, rivers and waterfalls.

Protozoan cysts such as amoeboids that were similar to *Entamoeba* sp. were detected in *C. brachyurus* and *L. guttulus* faeces. At Emas National Park in Goiás, Brazil, amoeboid cysts were also detected in the faeces of *C. brachyurus* [40]. Diagnosing these structures in faeces from free-living carnivores in a national park was an unexpected finding. However, this diagnosis needs to be reported, even if it might have resulted from contamination of the sample through contact with the soil or due to pseudoparasitism. The proximity of wild animals to humans, especially tourists visiting and camping in the park, may favour zoonotic transmission. In addition, *Balantioides coli* cysts were detected in the faecal material of artiodactyls in Itatiaia National Park, especially at Visconde de Mauá, where most samples from this group of animals were found. In addition to the macroscopic evaluation, the diagnosis of *B. coli* cysts contributed to confirming that these samples belonged to the order Artiodactyla. It is noteworthy that *S. scrofa* is considered the main reservoir for this protozoan, which has zoonotic transmission potential. Moreover, further surveys in environments proximate to human use should also be made in order to obtain information about the possibility of dissemination of B. coli between artiodactyls and humans.

The stabilization of the parasite accumulation curve demonstrated that the amount of samples collected from *C. brachyurus, L. gutullus* and *C. familiaris* was sufficient for all parasitological analyses, which is extremely important since it is not known how many individuals of each of these mammals inhabit the park. In Serra da Calçada, Minas Gerais, Brazil, faeces from *C. brachyurus* were found to present a lower richness index (R = 6) than that observed for the same species in the present study [28].

High parasite diversity was found for the faeces of *C. brachyurus* and *L. guttulus*, however few studies reported their diversity indices [26]. It is important to highlight that diversity indices, such as Shannon and Simpson’s indices, depend on the estimation of parasite abundance. Therefore, one of the major limitations of parasitological surveys through faecal analysis is the lack of an appropriate method for precisely quantifying the parasite abundancy since the number of eggs found in non-invasive faecal sampling may not reflect the actual parasite burden [41, 42].

Importantly, not all the parasites detected in faeces from *C. brachyurus* were present in samples from *C. familiaris*, and this may have given rise to significant parasite diversity in the pooled t test for canids. Although circulation of domestic dogs is prohibited in the park, they are sporadically seen on the trails, which increases the chances of this animal becoming infected with the same parasites as *C. brachyurus* and vice versa. Domestic animals have also been seen in or near other Brazilian conservation areas and are potential parasite dispersers [22, 25, 28, 35]. In this study, pets that were present in the park were associated with dog owners properties within or near the park, tourists or even abandonment; these are all situations which the park is not able to handle properly. Although the parasitic similarity between *C. brachyurus* and *C. familiaris* is low, it still denotes the possibility of parasite transmission between these canids, since all the structures detected in dog faeces were also detected in faeces from *C. brachyurus* and some positive samples from both canids were geographically proximate.

It needs to be borne in mind that canids, especially *C. brachyurus*, and *S. scrofa* are omnivorous. This dietary habit expands the feeding options of these animals, and this may have favoured infection by distinct parasitic agents in the present study. The parasite diversity and richness diagnosed in the felid faecal material, mainly in *L. guttulus*, may be directly related to carnivory. Thus, these animals can ingest a large variety of prey and are considered excellent parasite accumulators [43]. Since both felids and canids are territorial animals and travel long distances, they could also participate in the dispersal of parasites in the park environment.

It was also observed that the patterns of parasite structures in the faeces from *C. brachyurus* and *L. guttulus* were similar, as corroborated by the high Sorensen index. This similarity implies the possibility of shared parasites, since most of the samples from *C. brachyurus* were collected geographically proximate to the faeces of *L. guttulus*; both were mainly found in the upper part of Itatiaia National Park and in Visconde de Mauá (Figs. 1, 2 and 3).

In relation to *S. scrofa*, it was found that the accumulation curve for parasite structures did not stabilize, thus suggesting that the number of species could increase if more samples from these animals were collected. In faecal samples from the Serra da Capivara National Park, Piauí, Brazil, a similar picture to that in the present study was also seen with regard to both the richness and the non-stabilization of the *S. scrofa* graph maybe also because this species is omnivorous [25]. In addition, the different parasitism patterns observed between artiodactyls and carnivores seem to be associated with the specificity of the parasites to their hosts, the natural resources available in the park, the behaviour of the animal species (including their feeding habits) and the dispersal of parasite structures in different areas of Itatiaia National Park.

Importantly, some parasite taxa were shared among native, domestic (*C. familiaris*) and introduced (*S. scrofa*) animals. It was also verified that many of the detected parasites were zoonotic, including *Cryptosporidium* sp. and *Balantioides coli*. These species were mainly detected in the introduced species, especially in omnivorous animals that can move between the park and human dominated landscapes. Both the prospect of similar parasitic taxa between different hosts and that of zoonotic infectious agents should be faced with concern. Therefore, the parasites in introduced and domestic fauna should be frequently monitored, as they may have negative implications for wildlife conservation and even cause public health problems.

## Conclusions

Through the present study, it was possible to confirm the presence of mammalian species, such as carnivores and an artiodactyl, as well as the high richness and diversity of parasite structures in the faeces of these animals. Within this parasite richness, different helminth eggs, cysts, oocysts and protozoan antigens were detected. Our results demonstrated that the park has the elements necessary for the maintenance of complex parasite life cycles that include various hosts, such as intermediate and paratenic hosts. It is important to highlight that several parasites observed in the present study have the potential for zoonotic transmission, given that they may have been transmitted to animals due to their proximity to humans or due to some anthropogenic alterations. So, although it is a hard work, preventing domestic companion and invasive animals from entering and colonize the park would contribute to the maintenance of the environment balance in the park. Even so, the possibility that these parasites truly form part of the parasitic fauna of these animals cannot be ruled out. This scenario emphasizes the importance of constant surveillance of potentially infectious biological agents in the park and reserve environments.

## Methods

### Study site

**Fig. 8:**
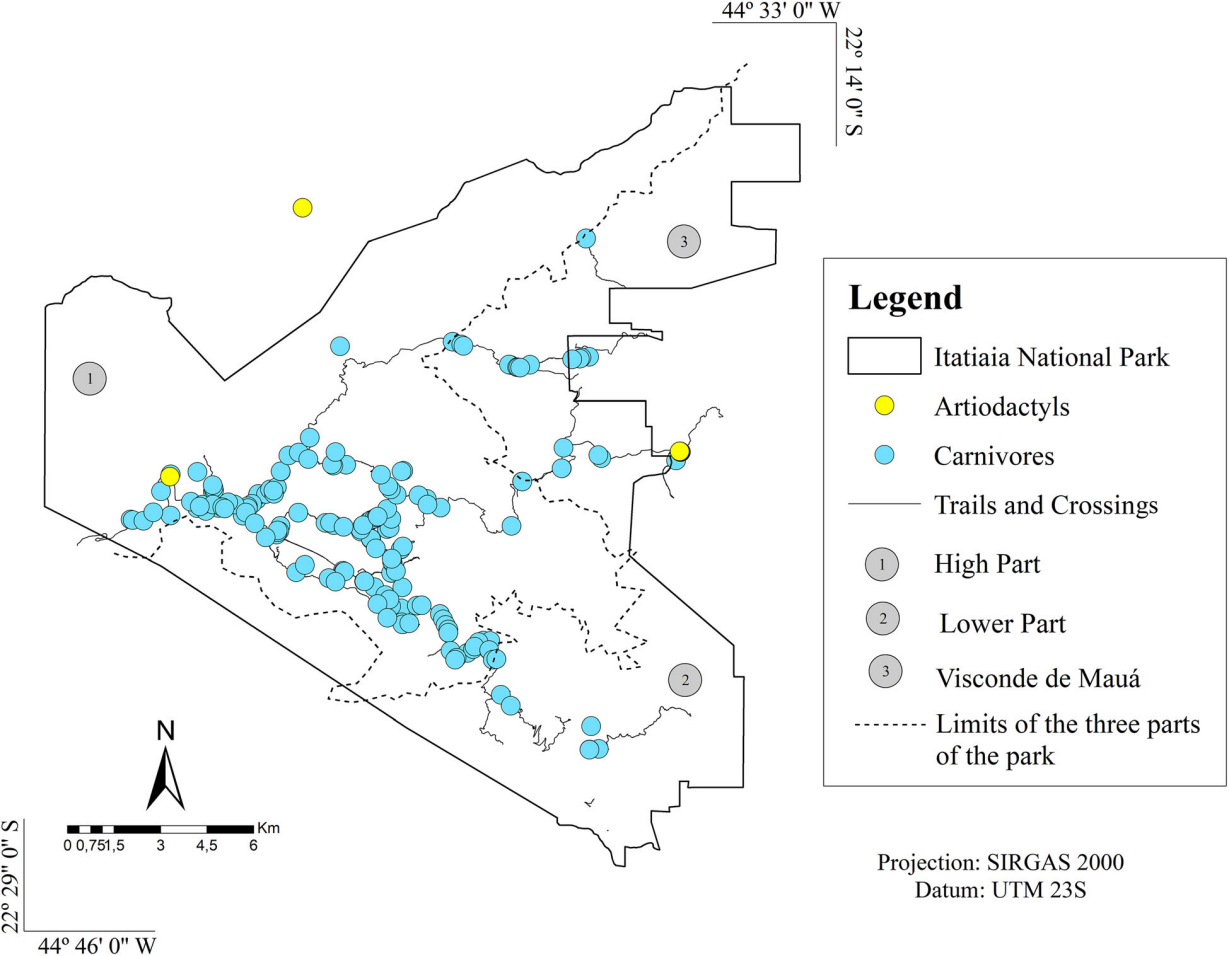
Sample collection points plotted on the map of Itatiaia National Park, Brazil (Arcgis version 10.5)

The study was carried out in Itatiaia National Park, which is a Brazilian protected area covering 28,084,100 ha. It is located in the Serra da Mantiqueira mountain range and encompasses parts of the states of Minas Gerais and Rio de Janeiro. The park is divided into three areas: the lower part, the high part and Visconde de Mauá. The Lower part encompasses the southern area of the park, where vegetation of the Atlantic Forest biome predominates. The high part, where the Maciço das Prateleiras and the Agulhas Negras are located, comprises rock formations and predominantly high-altitude grassland vegetation, and Visconde de Mauá, which has predominantly Atlantic Forest vegetation and many waterfalls (Fig. 8).

Itatiaia National Park is located in the Atlantic Forest biome and presents a wide range of abiotic factors, such as different types of soil, atmospheric pressure, and temperatures. The relief is mainly mountainous, and the elevation ranges from 540 m in the southern part of the park to 2791.55 m at Pico das Agulhas Negras. The climate is moderate and humid, with temperatures ranging from 10 °C to 18 °C [44]. The park also has a high diversity of biotic factors, including plants, animals, microorganisms and helminths. Regarding biodiversity, many animal species have been catalogued in this park, including reptiles, amphibians, mammals and birds. This includes 111 species of mammals that have been identified.

### Collection of faecal samples

Between June 2017 and April 2018, faecal material was obtained through opportunistic sampling. This material was only collected if it was morphologically consistent with the faeces of carnivores or artiodactyls. Faecal samples that were extremely dehydrated and/or deteriorated were not collected. A total of 352.2 km was surveyed, including 27 trails, 3 crossings and 6 roads. In addition, a sample from a georeferenced artiodactyl outside the park in the municipality of Campo Redondo, Minas Gerais, was also collected. Since this specimen was caught on a trail leading to the park, its sample was also included in this study. During field collection, all samples were georeferenced, identified with the aid of identification keys, photographed, and stored in plastic bags without chemical preservatives in non-refrigerated bags. In addition, the identification number, date, time, and place of collection were registered on each datasheet for each sample. All obtained faeces were sent to the Laboratory of Parasitology at the Biomedical Institute of Fluminense Federal University, where they were refrigerated for 2 days.

### Host identification - macroscopic morphological analysis

The first step in identifying the host species was macroscopic morphological analysis of the stool samples. First, the samples were weighed and then the material was deposited on a white sheet to register the coloration and presence of artefacts and dietary components and measured. After, all this information was compared with the faecal morphology descriptions of the mammalian species of Brazil [12, 45].

### Host identification - guard hair trichology

To retrieve any hair present in the collected faecal samples, half of each sample was washed, dried and stored in plastic bags. The guard hairs were then selected and subjected to cuticular impression and medullary diaphanization [46]. The cuticular and medullary patterns of the guard hairs were examined, photomicrographed using an Olympus® BX 41 optical microscope, and compared with descriptions in the literature [46–56]. In addition, reference slides were made using guard hairs retrieved from mammal faeces collected at the Rio de Janeiro Zoo, guar hairs deposited in collections in the Serra dos Órgãos National Park and hairs from taxidermized animals from Itatiaia National Park.

### Host identification – DNA sequencing

#### Faecal sample preprocessing and DNA extraction

The second half of the faecal sample was homogenized in distilled water, and the resulting filtrate was aliquoted into 15 mL conical-bottom centrifuge tubes, which were refrigerated for 3 to 4 days and subjected to faecal suspensions and DNA extraction.

The faecal suspensions were then prepared in sterile tubes using 200 μl of faecal filtrate and 800 μl of 0.01 M Tris-Ca^++^ buffer (pH 7.2). After centrifugation at 1500 RPM for 10 min, the supernatant was collected and transferred to another sterile tube, where 100 μL of chloroform was added. After another centrifugation, the resulting faecal suspension was collected and aliquoted into 1.5 mL microtubes, which were stored at − 20 °C overnight. DNA extraction was then performed from 200 μl of the faecal suspension using the High Pure PCR Template Preparation kit (Roche®) following the manufacturer’s recommendations.

#### Polymerase chain reaction (PCR), sequencing and phylogenetic analysis

PCR was performed on the carnivore faecal samples using the forward primer Car12Ss2 (5 ‘GGTTTGGTCCTRGCCTT 3’) and the reverse primer Car12Ss2 (5 ‘AGCAAGGTGTTATGAGCTAC 3’), which amplify a 12S mitochondrial gene fragment [57]. Samples that presented low-quality electropherograms were also submitted to PCR using the forward primer ATP6-DF3 (5 ‘AACGAAAATCTATTCGCCTCT 3’) and reverse primer ATP6-DR1 (5 ‘CCAGTATTTGTTTTGATGTTAGTTG 3’), which amplify a fragment of the ATP6 mitochondrial gene. To analyse the samples from the artiodactyls, the forward primer BC-F2 (5 ‘ATCACCACTATTGTTAATATAAAACC 3’) and reverse primer HCO2198 (5 ‘TAAACTTCAGGGTGACCAAAAAATCA 3’) were used to amplify a fragment of the COI mitochondrial gene. Both PCRs were performed using validated protocols [58]. All amplified products were confirmed through electrophoresis on a 1.5% agarose gel and were purified using the ExoSAP-IT enzyme and sequenced in the forward direction by a 3730 × 1 DNA Analyser automated sequencer (Applied Biosystems). Finally, the sequences were aligned with the reference sequences, which were retrieved from GenBank, using BioEdit software, version 7.2.5. The DNA sequences from the mammalian samples matched the DNA reference sequences at a level of 95% or higher (Table 6).

**Table 6:** Similarity, accession number, and publication of genic reference sequences used for classification of the hosts

Species	12S			ATP6			COI		
	% identity	References	References obtained from this study	% identity	References	References obtained from this study	% identity	Reference	References obtained from this study
*Chrysocyon brachyurus*	98.04–100%	KJ508409	MN509185	100%	KJ508409	10.5061/dryad.djh9w0vvx	–	–	–
		MF802260	MN509186		–				
			MN509187						
			MN509188						
			MN509189						
			MN509190						
			MN509191						
*Cerdocyon. thous*	100%	MN444854	MN509192	–	–	–	–	–	–
*Canis familiaris*	98.04–100%	MH746950	MN509193	–	–	–	–	–	–
		KT591870	MN509194						
*Leopardus guttulus*	95.16–100%	MF802256	MN509195	96.3–100%	–	10.5061/dryad.djh9w0vvx	–	–	–
			MN509196						
			MN509197						
			MN509198						
*Puma concolor*	–	–	–	100%	MH818222	10.5061/dryad.djh9w0vvx	–	–	–
					MH814705				
*Sus scrofa*	–	–	–	–	–	–	96.43–100%	MG837550	MN608174
									MN608175
									MN608176

### Parasitological techniques - microscopy

The filtrate resulting from the second half of each sample was used for faecal sample preprocessing and DNA extraction and for parasitological techniques. The filtrate was aliquoted into 15 mL conical-bottom centrifuge tubes, which were subjected to centrifuge-sedimentation [59, 60], centrifugal-flotation techniques using zinc at a density of 1.180 g/cm^3^ [61] and centrifugal flotation with 1.300 g/cm^3^ sucrose solution [62, 63]. The remaining filtrate was transposed to a conical-bottom glass for use with the spontaneous sedimentation technique [64]. The microscopy slides obtained from each parasitological technique were read and photomicrographed using an Olympus® BX 41 optical microscope; slides were initially examined at 100X magnification and, when necessary, at 400X magnification. The morphometry of the parasite structures was evaluated using a 400X and 1000X magnification eyepiece under an Olympus® BX 41 microscope.

### Parasitological techniques - ELISA for Cryptosporidium sp.

The frozen samples in microtubes were subjected to enzyme-linked immunosorbent assay (ELISA) using the “*Cryptosporidium* antigen detection microwell” kit (IVD Research®). Prior to the enzyme immunoassay, a solution was made using 60 μL of the sample and 60 μL of the diluent provided in the kit. After dilution, 100 μL of this solution was transferred to the assay plate, and thus, the technique was performed as recommended by the manufacturer. The plates were read in an ELISA reader (Thermo plate® TP-reader LGC Biotechnology Ltda.).

### Analysis of results

The host identification was summarized with the parasitological results. Since the techniques did not provide information with the same degree of precision for the host taxonomy, an association was made between the more specific results of each method and the final classification of the host (Table 7). Pearson’s correlation coefficient (ρ) was calculated to support the association of information for the identification of host species. This method was not used for those samples for which all three identification techniques completely agreed.

**Table 7:** Criteria used in the association of information obtained from macroscopic analysis of faeces, trichology of guard-hair and DNA sequencing for the final classification of hosts

Host identification	
Host species	
1) Association of the 3 techniques when they completely agreed with each other.	
2) Association of 2 techniques when one of the methods does not provide taxonomic information about the host.	
3) Trichology + DNA sequencing when macroscopy provides taxonomic information that does not agree with the obtained by other techniques.	
4) Macroscopy + DNA sequencing DNA when: - Trichology provided information on small carnivores (mustelids and procionids), which are incompatible with the morphology of samples with large fecal volume. - Different species of small felids were identified by trichology and sequencing.	
Unindentified species of Carnivores / Artiodactyls	
1) Complete disagreement with all information obtained by the techniques.	
2) Taxonomic information from the host obtained only by a single identification technique.	
3) Identification of a feline and a canine by trichology and sequencing.	
4) Absence of information on gene sequencing and information on small carnivores (mustelids and procionids) by trichology, which are incompatible with the morphology of samples with large fecal volume.	
5) Absence of taxonomic information on the host species.	

Through macroscopy, the samples were classified into a taxonomic group of order or family. Using trichology, the samples were classified into a family and species taxonomic group, and DNA sequencing provided taxonomic information about the host species.

Faecal samples were considered positive for gastrointestinal parasite structures when at least one cyst, oocyst, egg or nematode larva was detected and/or antigen of *Cryptosporidium* sp. was shown. The parasitological results were presented descriptively at the lowest possible taxonomic rank and into morphotypes of helminth eggs and protozoan oocysts. These morphotypes were distinguished from each other by their taxonomic rank, morphology (colour and shape) and size. The richness, diversity, and similarity indices and Principal Component Analysis (PCA) were analysed only according to the results obtained from the different taxa parasite structures. The morphotypes detected in the same taxa were not considered in the index analysis.

These statistical tests were performed using Past® software, version 3.2.2 [65]. The parasite richness for each host species was determined by counting the different taxa detected in its samples; in addition, the sample sufficiency was plotted on accumulation curves of the parasite species [66].

The parasite diversity was analysed statistically using the Shannon (H′) and Simpson© indices [67]. The statistical significance of Shannon’s diversity for the parasites was analysed using a pooled t test, with a significance level of 5% [68]. Sorensen’s index (S) was also used to compare the similarity of the parasite structures among host species [67].

The Shannon and Simpson diversity indices for the parasites were complementary and were analysed to verify the relative abundances of the species for the set of samples of each host species. The highest parasitic diversity was determined in the hosts that presented high Shannon index and Simpson index values of close to 1.

## Supplementary information

**Additional file 1 S1 Table.** Macro and microscopic morphology of guard hairs and frequency of mammalian taxa identified by trichology in faecal samples collected in Itatiaia National Park, Brazil.

## Data Availability

The datasets generated in this study from AT6 region are available in the Dryad repository under DOI number 10.5061/dryad.djh9w0vvx. The other sequences are available in GenBank-NCBI under accession numbers MN509185, MN509186, MN509187, MN509188, MN509189, MN509190, MN509191, MN509192, MN509193, MN509194, MN509195, MN509196, MN509197, MN509198 for 12S gene and MN608174, MN608175, and MN608176 for COI.

## Abbreviations

*C. brachyurus*: *Chrysocyon brachyurus*
*C. familiaris*: *Canis familiaris*
*C. thous*: *Cerdocyon thous*
ELISA: Enzyme-Linked Immunosorbent Assay
*L. guttulus*: *Leopardus guttulus*
*L. pardalis*: *Leopardus pardalis*
PCA: Principal Components Analysis
*P. concolor*: *Puma concolor*
PNI: Itatiaia National Park
PCR: Polymerase chain reaction
*P. yagouaroundi*: *Puma yagouaroundi*
*S. scrofa*: *Sus scrofa*
Syn: Synonymous
UPGMA: Unweighted Pair Group Method with Arithmetic Mean
US: Unidentified Species

## References

[1] Wilson DE, Mittermeier RA (2009). The mammals of the world.

[2] Aranda RC, Serrano-Martínez E, Tantaleán VM, Quispe HM, Casas VG (2013). Identificación y frecuencia de parásitos gastrointestinales en félidos silvestres en cautiverio en el Perú. Rev Investig Vet Perú.

[3] Poulin R (1999). The functional importance of parasites in animal communities: many roles at many levels?. Int J Parasitol.

[4] Freeland WJ (1983). Parasites and the coexistence of animal host species. Am Nat.

[5] Azpiri GS, Maldonado FG, González GC (2000). La importancia del estudio de enfermedades en la conservación de fauna silvestre. Vet México.

[6] Barutzki D, Schaper R (2003). Endoparasites in dogs and cats in Germany 1999-2002. Parasitol Res.

[7] Daszak P (2000). Emerging infectious diseases of wildlife- threats to biodiversity and human health. Sci..

[8] Emmons LH, Feer F (1997). Neotropical rainforest mammals, a field guide.

[9] Cleveland S, Hess GR, Dobson MK, Laurenson HI, McCallum MG, Roberts MG, Woodroffe R, Hudson PJ, Rizzoli A, Grenfell BT, Heesterbeek H, Dobson AP (2002). The role of pathogens in biological conservation. The Ecology of Wildlife Diseases.

[10] Morin PA, Woodruff DS, Smith TB, Wayne RK (1996). Noninvasive Genotyping for Vertebrate. Molecular genetic approaches in conservation.

[11] Taberlet P, Waits LP, Luikart G (1999). Noninvasive genetic sampling: look before you leap. Trends Ecol Evol.

[12] Chame M (2003). Terrestrial mammal feces: a morphometric summary and description. Mem Inst Oswaldo Cruz.

[13] Dib LV, Palmer JPS, Lima CSCL, Bastos OMP, Uchôa CMA, Amendoeira MRR, Bastos ACMP, Barbosa AS, Baker A (2019). Noninvasive Sampling: Monitoring of Wild Carnivores and Their Parasites. Protected Areas, National Parks and Sustainable Future.

[14] Calixto JB, Siqueira JM (2008). The Drug Development in Brazil: Challenges. Gazeta Médica da Bahia.

[15] ICMBio Instituto Chico Mendes de Conservação da Biodiversidade 2016. Carnívoros brasileiros. http://www.icmbio.gov.br/cenap/carnivoros-brasileiros.html. Accessed 22 Apr 2019.

[16] Dib LV, Cronemberger C, Pereira FA, Bolais PF, Uchôa CMA, Bastos OMP, Amendoeira MRR, Barbosa AS (2018). Gastrointestinal parasites among felids inhabiting the Serra dos Órgãos National Park, Rio de Janeiro. Brazil. Rev Bras Parasitol Vet..

[17] Seton ET (1925). On the study of scatology. J Mamm.

[18] Silva-Caballero A (2010). Parasitosis gastrointestinales en felinos silvestres en Nanchititla, México.

[19] Contreras MG (2014). Parásitos gastrointestinales de felinos de la Reserva Ecológica El Edén A.C. Quintana Roo, México.

[20] Solórzano-García B, White-Day JM, Gómez-Contreras M, Cristóbal-Azkárate J, Osorio-Sarabia D, Rodríguez-Luna E (2017). Coprological survey of parasites of free-ranging jaguar (*Panthera onca*) and puma (*Puma concolor*) inhabiting 2 types of tropical forests in Mexico. Rev Mex Biodivers.

[21] Beltrán-Saavedra LF, Angulo S, Gonzales JL (2009). Uso de metodologías de censos muestrales indirectos de fecas para evaluar endoparásitos en mamíferos silvestres: Un ensayo en la Reserva Privada de San Miguelito, Santa Cruz. Bolívia Ecol Biol.

[22] Araujo RDS (2014). Enteroparasitos de carnívoros silvestres e *Canis familiaris* (Linnaeus 1758) (Mammalia; Carnivora) na Reserva Particular do Patrimônio Natural Santuário do Caraça, Minas Gerais.

[23] Srbek-Araujo AC, Santos JLC, Almeida VM, Guimarães MP, Chiarello AG (2014). First record of intestinal parasites in a wild population of jaguar in the Brazilian Atlantic Forest. Rev Bras Parasitol Vet.

[24] Patton S, Rabinowitz AR (1994). Parasites of wild Felidae in Thailand: a Coprological survey. J Wild Dis.

[25] Fiorello CV, Robbins RG, Maffei L, Wade SE (2006). Parasites of free-ranging small canids and felids in the Bolivian Chaco. J Zoo Wild Med.

[26] Brandão ML, Chame M, Cordeiro JLP, Chaves SAM (2009). Diversidade de helmintos intestinais em mamíferos silvestres e domésticos na Caatinga do Parque Nacional Serra da Capivara, Sudeste do Piauí. Brasil Rev Bras Parasit Vet.

[27] Kusma SC, Wrublewski DM, Teixeira VN, Holdefer DR (2015). Parasitos intestinais de *Leopardus wiedii* e *Leopardus tigrinus* (Felidae) da Floresta Nacional de Três Barras, SC. Luminária.

[28] Massara R, Paschoal A, Chiarello A (2015). Gastrointestinal parasites of maned wolf (*Chrysocyon brachyurus*, Illiger 1815) in a suburban area in southeastern Brazil. Braz J Biol.

[29] Wrublewski DM, Kusma SC, Teixeira VN (2018). Parasitos gastrointestinais em *Puma concolor*, *Puma yagouaroundi* e *Leopardus pardalis* (Carnivora: Felidae) na Floresta Nacional de Três Barras, SC, Brasil. Rev Acad Cien Anim.

[30] Rodriguez-Rivera LD, Cummings KJ, McNeely I, Suchodolski JS, Scorza AV, Lappin MR, Mesenbrink BT, Leland BR, Bodenchuk MJ (2016). Prevalence and diversity of *Cryptosporidium* and *Giardia* identified among feral pigs in Texas. Vector-Borne and Zoonotic Dis.

[31] Conboy G (2009). Cestodes of dogs and cats in North America. Vet Clin North Am Small Anim Pract.

[32] Souza HCD (2014). Helmintos intestinais de Tayassuidae e Suidae (Mammalia: Artiodactyla) no Pantanal: um estudo sobre a circulação de espécies na Reserva Particular do Patrimônio Nacional SESC Pantanal e seu entorno, Barão de Melgaço.

[33] Ruas JL, Muller G, Farias NAR, Gallina T, Lucas ASG, Pappen F, Sinkoc AL, Brum JGW (2008). Helmintos do cachorro do campo, *Pseudalopex gymnocercus* (Fischer, 1814) e do cachorro do mato, Cerdocyon thous (Linnaeus, 1766) no sul do estado do Rio Grande do Sul. Brasil Rev Bras Parasitol Vet.

[34] Lindsay DS, Dubey JP, Blagburn BL (1997). Biology of *Isospora* spp. from humans, nonhuman primates, and domestic animals. Clin Microbiol Ver.

[35] Santos JLC, Magalhães NB, Santos HA, Ribeiro RR, Guimarães MP (2008). Parasites of domestic and wild canids in the region of Serra do Cipó National Park, Brazil. Rev Bras Parasitol Vet.

[36] Šlapeta J (2013). Cryptosporidiosis and *Cryptosporidium* species in animals and humans: a thirty colour rainbow?. Int J Parasitol.

[37] Holubová N, Sak B, Horčičková M, Hlásková L, Květoňová D, Menchaca S, McEvoy J, Kváč M (2016). *Cryptosporidium avium* n. sp. (Apicomplexa: Cryptosporidiidae) in birds. Parasitol Res.

[38] Kváč M, Havrdová N, Hlásková L, Daňková T, Kanděra J, Ježková J, Vítovec J, Sak B, Ortega Y, Xiao L, Modrý D, Chelladurai JR, Prantlová V, McEvoy J (2016). *Cryptosporidium proliferans* n. sp. (Apicomplexa: Cryptosporidiidae): molecular and biological evidence of cryptic species within gastric *Cryptosporidium* of mammals. PLoS One.

[39] Zahedi A, Paparini A, Jian F, Robertson I, Ryan U (2016). Public health significance of zoonotic *Cryptosporidium* species in wildlife: critical insights into better drinking water management. Int J Parasitol: Paras Wild.

[40] Braga RT, Vynne C, Loyola RD (2012). Fauna parasitária intestinal de *Chrysocyon brachyurus* (lobo-guará) no Parque Nacional das Emas. Bioikos..

[41] Gillespie TR (2006). Noninvasive assessment of gastrointestinal parasite infections in free-ranging primates. Int J Primatol.

[42] Greiner EC, McIntosh A, Huffman MA, Chapman CA (2009). Collection methods and diagnostic procedures for primate parasitology. Primates parasite ecology. The dynamics and study of host–parasite relationships.

[43] Rocha FL (2013). A rede trófica e o papel dos carnívoros silvestres (Ordem Carnivora) nos ciclos de transmissão de *Trypanosoma cruzi*.

[44] ICMBio - Instituto Chico Mendes de Conservação da Biodiversidade. 2013. Plano de Manejo do Parque Nacional do Itatiaia. Encarte 3. Available on : <http://www.icmbio.gov.br/portal/component/content/article?id=2181:parna-do-itatiaia>. Access realized at: 11 Apr 2020.

[45] Borges PL, Tomás WM. Guia de rastros e outros vestígios de mamíferos do Pantanal. 1st ed. Pantanal com Ciência, SEBRAE MS: Mato Grosso do Sul; 2008.

[46] Quadros J (2002). Identificação microscópica de pêlos de mamíferos brasileiros e sua aplicação no estudo da dieta de carnívoros.

[47] Hess WM, Flinders JT, Pritchett CL, Allen JV (1985). Characterization of hair morphology in families Tayassuidae and Suidae with scanning Electron microscopy. J Mamm..

[48] Teerink BJ (1991). Hair of west European mammals: atlas and identification.

[49] Martins IA. Identificação dos canídeos brasileiros através dos seus pêlos guarda: Paulista State University; 2005. http://rgdoi.net/10.13140/RG.2.1.3596.1760. Accessed 26 June 2019.

[50] Fernandes MAW (2008). Análise comparativa da morfologia dos pelos-guarda de mamíferos com hábito semi-aquático.

[51] Vanstreels RET, Ramalho FP, Adania CH (2010). Microestrutura de pêlos-guarda de felídeos Brasileiros: considerações para a identificação de espécies. Biota Neotrop..

[52] Abreu MSL, Christoff AU, Vieira EM (2011). Identificação de marsupiais do Rio Grande do Sul através da microestrutura dos pelos-Guarda. Biota Neotrop..

[53] Duarte TDS (2013). Micromorfologia de pelos aristiformes de roedores das famílias Cricetidae e Echimyidae (Mammalia, Rodentia).

[54] Silveira F, Sbalqueiro IJ, Monteiro-Filho ELA (2013). Identificação das espécies brasileiras de *Akodon* (Rodentia: Cricetidae: Sigmodontinae) através da microestrutura dos pelos. Biota Neotrop.

[55] Miranda GHB, Rodrigues FHG, Paglia AP (2014). Guia de Identificação de Pelos de Mamíferos Brasileiros.

[56] Alberts CC, Saranholi BH, Frei F, Galetti PM (2017). Comparing hair-morphology and molecular methods to identify fecal samples from Neotropical felids. PLoS ONE.

[57] Rodríguez-Castro KG, Saranholi BH, Bataglia L, Blanck DV, Galetti PM Jr. Molecular species identification of scat samples of south American felids and canids. Conserv Genet Resour. 2018:1–6. 10.1007/s12686-018-1048-6.

[58] Chaves PB, Graeff VG, Lion MB, Oliveira LR, Eizirik E (2012). DNA barcoding meets molecular scatology: short mtDNA sequences for standardized species assignment of carnivore noninvasive samples: carnivore dna mini-barcodes. Mol Ecol Resour.

[59] Ritchie LS (1948). An ether sedimentation technique for routine stool examinations. Unit S Arm Med Depart Bull.

[60] Young KH, Bullock SL, Melvin DM, Spruill CL (1979). Ethyl Acetate as a Substitute for Diethyl Ether in the Formalin-Ether Sedimentation Technique. J Clin Microbiol.

[61] Faust EC, D’Antoni JS, Odon V, Miller MJ, Perez C, Sawitz W, Thomen F, Tobie JE, Walker JH (1938). A critical study of clinical laboratory techniques for the diagnosis of protozoan cysts and helminth eggs in feces. I. Preliminary communication. Am J Trop Med.

[62] Sheather AL (1923). The detection of intestinal protozoa and mange parasites by a floatation technique. J Comp Pathol Therap.

[63] Huber F, Bomfim TC, Gomes RS (2003). Comparação da eficiência da técnica de sedimentação pelo formaldeído-éter e da técnica de centrífugo-flutuação modificada na detecção de cistos de *Giardia* sp. e oocistos de *Cryptosporidium* sp. em amostras fecais de bezerros. Rev. Bras. Parasitol. Vet..

[64] Lutz A (1919). O Schistosomum mansoni e a schistosomatose segundo observações feitas no Brasil. Mem Inst Oswaldo Cruz.

[65] Hammer (2019). Past version 3.25 Reference manual. Paleontological Statistics.

[66] Colwell RK, Coddington JA (1994). Estimating terrestrial biodiversity through extrapolation. Phil Trans R Soc Lond B.

[67] Krebs CJ (1999). Ecological Methodology.

[68] Poole RW (1974). An introduction to quantitative ecology.

