# Supplementary material for: Non-invasive sampling in Itatiaia National Park, Brazil: wild mammal parasite detection
Source: BMC Vet Res. 2020 Aug 17;16:295. doi: 10.1186/s12917-020-02490-5 (PMC7430008; doi:10.1186/s12917-020-02490-5)
Supplement: Supplementary file 1 — Additional file 1 S1 Table. Macro and microscopic morphology of guard hairs and frequency of mammalian taxa identified by trichology in faecal samples collected in Itatiaia National Park, Brazil. [file 12917_2020_2490_MOESM1_ESM.docx]

* No cuticle was observed, only fibers with bundles of cells arranged parallel to each other in the direction of hair length

** > 2/3 of the hair width

*** < 2/3 of the hair width


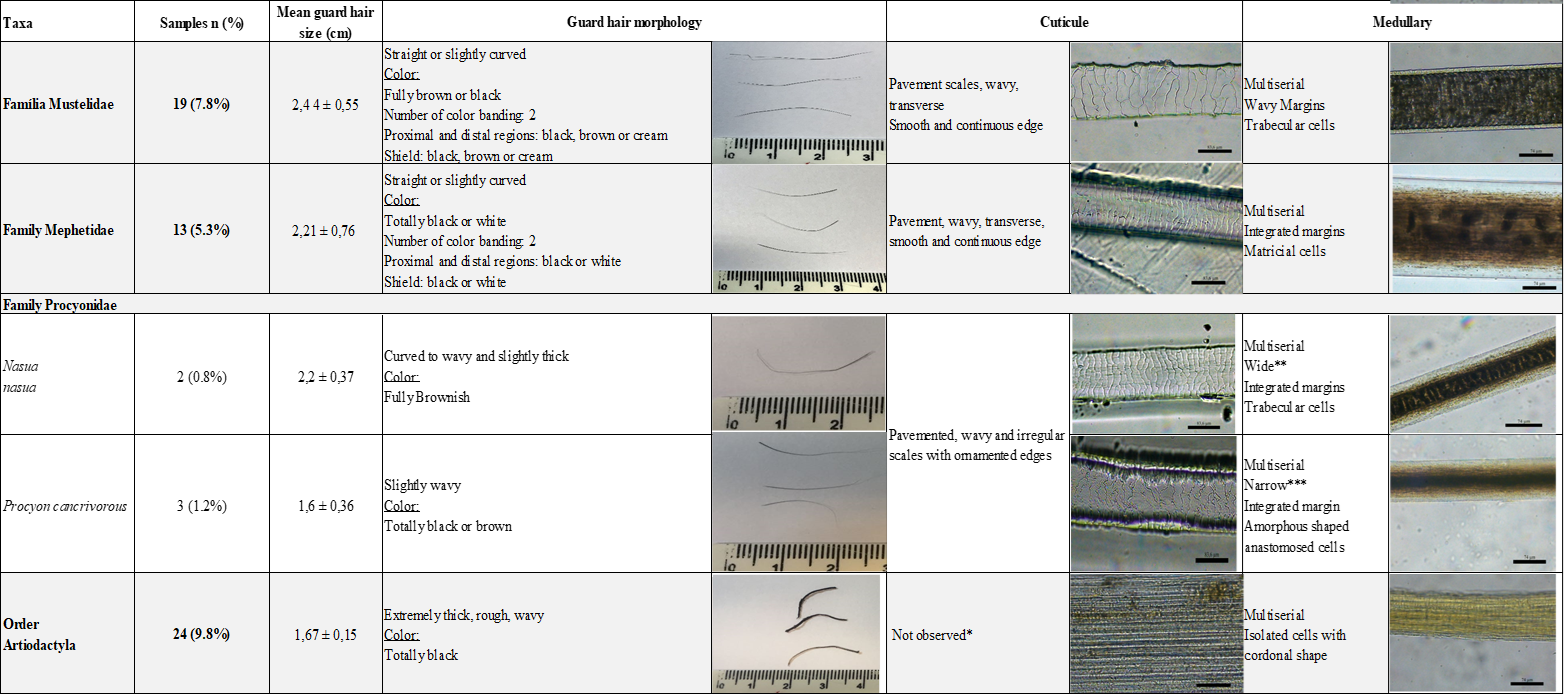


* No cuticle was observed, only fibers with bundles of cells arranged parallel to each other in the direction of hair length

** > 2/3 of the hair width

*** < 2/3 of the hair width
